# Editorial: Discourse, conversation and argumentation: theoretical perspectives and innovative empirical studies, volume IV

**DOI:** 10.3389/fpsyg.2026.1827630

**Published:** 2026-04-08

**Authors:** Antonio Bova, Francesco Arcidiacono, Carlo Galimberti, Lise Haddouk

**Affiliations:** 1 Department of Psychology, Catholic University of the Sacred Heart, Milan, Italy; 2 Centro Studi e Ricerche di Psicologia della Comunicazione (PSICOM), Catholic University of the Sacred Heart, Milan, Italy; 3 Research Department, Haute Ecole Pédagogique – Cantons de Berne Francophone, Jura et Neuchâtel, Biel/Bienne, Switzerland; 4 Department of Human and Social Sciences, University “Mercatorum”, Rome, Italy; 5 Department of Psychology, Université de Rouen, Mont-Saint-Aignan, France

**Keywords:** argumentation, communicative interactions, conversation, discourse, psychology

Despite the central role of discourse, conversation, and argumentation in social life, psychological research still lacks a unified framework for understanding these communicative phenomena across contexts, methods, and theoretical traditions. Investigations of language use, dialogic interactions, and argumentative processes continue to develop within partially separate research communities, often limiting the possibility of a systematic interdisciplinary exchange.

This fourth volume of the Research Topic “*Discourse, conversation and argumentation: theoretical perspectives and innovative empirical studies*” aims to further bridge this divide by bringing together innovative contributions that examine communicative practices from a broad psychological and socio-discursive perspective. The papers collected in this Research Topic explore the place of discourse within institutional, educational, interpersonal, media, and intercultural contexts, while also advancing a methodological reflection through corpora analyses, meta-analyses, comparative discourse analyses, stories completion, and functional linguistic approaches.

Taken together, the contributions presented in this volume demonstrate the vitality and diversity of contemporary research on communication: they show how discourse and interaction are deeply implicated in the construction of identities, values, institutional positions, epistemic stances, and moral responsibilities. At the same time, they reveal how communicative practices are shaped by historical changes, cultural contexts, technological environments, and shifting social norms. By assembling studies that range from media representations and diplomatic discourses to educational dialogues, research writings, executive identities, and digital subtitlings, this volume offers a rich and multifaceted account of the psychological significance of language in use.

Zhang opens the volume with a diachronic corpus-based investigation of the construction of Henan province's regional image in Western mainstream media. Combining text mining with transitivity-based social role analysis, the study traces how semantic themes and role configurations have changed over time. The findings reveal a shift from a predominantly crisis-oriented and goal-positioned representation toward a more agentive construction in which Henan increasingly appears as actor, thereby illustrating how discourse contributes to the historical reconfiguration of regional identity.

Rühlemann addresses one of the foundational questions of conversation research: how speakers recognize that a turn is nearing completion. Drawing on the Freiburg multimodal interaction corpus, the study explores whether frequency and frequency-related measures can function as cues for turn completion. By comparing question turn-constructional units with storytelling units, the paper shows how word-frequency patterns may help listeners anticipate turn transition, thus offering an original contribution to the study of turn-taking as a socially organized and cognitively informed process.

Xing et al. examine the effects of interactive reading on young children's narrative abilities through a meta-analysis of 25 studies. Their findings show a medium positive overall effect of interactive reading on narrative development, while also identifying intervention duration and peer sharing as important moderators. The study provides robust quantitative evidence for the educational value of interactive reading and highlights the relevance of dialogic practices in supporting children's communicative and developmental trajectories.

Schwarz et al. focus on ethical learning in educational dialogue. Using the analytical distinction between dialogue on ethics and ethics of dialogue, their case study illuminates the conditions under which ethical thinking and ethical conduct can reinforce one another in classroom interaction. Their analysis shows that the coordination of epistemic and behavioral dimensions of ethical learning depends crucially on dialogic design principles, thereby contributing to research on democratic education and values-oriented interaction.

Zheng offers a cross-lingual analysis of attitudinal meaning in the publicity discourses of Anglo-American and Chinese universities. Drawing on the appraisal system, the study reveals significant differences in the distribution of affective, judgmental, and appreciative resources across the two linguistic and cultural contexts. The findings show how university discourse encodes distinct communicative priorities, with implications not only for cross-cultural discourse research but also for institutions seeking to strengthen their global communication strategies.

Yang and Guo examine changing patterns of epistemic positioning in research writing across education, history, mechanical engineering, and physics over three historical periods. Their analysis of hedges and boosters reveals a longitudinal decrease in overt epistemic positioning, suggesting a broader shift toward a more impersonal and data-oriented scientific style. This study contributes to our understanding of how disciplinary discourse evolves over time and how knowledge claims are rhetorically calibrated in changing academic cultures.

Liu turns attention to the discursive construction of scholar identity through a critical genre analysis of Chinese and English academic biographies. By combining textual analysis with interview data, the study identifies both common rhetorical structures and culturally specific identity strategies. The paper demonstrates how academic selves are constructed not only through genre conventions but also through institutional and socio-cultural expectations, offering valuable insights into identity work in academic communication.

Li and Pan analyze the discursive strategies used by spokespersons of China's ministry of foreign affairs during a public health crisis. Their corpus-driven study shows how communicative, offensive, and juxtapositional strategies were employed to articulate national positions, counter criticism, and construct geopolitical alignments. The paper highlights the role of diplomatic discourse in managing crisis narratives and shaping international legitimacy.

Zeng explores danmu subtitling on Bilibili as a self-regulative and ethically complex translation practice. By extending Chesterman's models of translation ethics and proposing an ethics of digital technology, the study shows how user-generated subtitles are governed by multiple and sometimes competing ethical principles. This research makes an important contribution to the study of digital participatory discourse, translation practices, and the ethical dimensions of collaborative online communication.

Manca et al. investigate how executive managers in Italy and the United Kingdom narratively reconfigured the self in the wake of COVID-19. Using the story completion method, the authors identify alternative self-definitions emerging at the intersection of work, family, and cultural expectations. The study shows how major societal disruptions can open new identity trajectories, including becoming a work-life balance advocate, a family man, or a renewed ideal worker.

Finally, Xiang examines the Mandarin construction “Taishang zuo-zhe zhuxituan” through the lens of Cardiff grammar. The study clarifies the process type, participant roles, syntactic structure, and discourse-functional motivations of this construction, showing how grammatical analysis can illuminate the relationship between linguistic form and communicative function. In doing so, it contributes to a deeper understanding of Mandarin grammar and to broader functional approaches to discourse.

The studies collected in this fourth volume offer compelling evidence of the breadth, complexity, and methodological richness of current research on discourse, conversation, and argumentation. Whether focusing on interactional cues, educational dialogue, academic identity, media representation, diplomatic communication, or digital translation, these contributions show that communicative practices are central to the ways individuals and institutions construct meaning, negotiate values, and respond to social change. We hope that this volume will stimulate further interdisciplinary dialogue and inspire new research at the intersection of psychology, discourse studies, and communication.

